# Supplementary material for: MetaRibo-Seq measures translation in microbiomes
Source: Nat Commun. 2020 Jun 29;11:3268. doi: 10.1038/s41467-020-17081-z (PMC7324362; doi:10.1038/s41467-020-17081-z)
Supplement: Supplementary file 10 — Supplementary Data 7 [file 41467_2020_17081_MOESM10_ESM.zip › File2/Confidence_High_Taxonomy/420850_out.krona.html]

Javascript must be enabled to view this page.

members
magnitude
magnitudeUnassigned
count
unassigned
taxon
rank

420850\_out

297

297
2
superkingdom

297
phylum
976

200643
class
297

297
order
171549

297
family
815

297
816
genus


SRS019286\_contig\_number\_4068SRS020869\_contig\_number\_13371SRS104636\_contig\_number\_26073SRS147139\_contig\_number\_31209
4
species
1263049


SRS1054691\_contig\_number\_12478
1
2291999
species


SRS024388\_contig\_number\_2083
1
species
671267

species
291644

SRS053649\_contig\_number\_11378
1

species
47678

SRS142890\_contig\_number\_4883
1

species
246787
89

SRS013476\_contig\_number\_32599SRS014235\_contig\_number\_39774SRS014287\_contig\_number\_contig-100\_1497.107149SRS014459\_contig\_number\_35096SRS014979\_contig\_number\_33409SRS015065\_contig\_number\_14846SRS015190\_contig\_number\_11253SRS015431\_contig\_number\_75518SRS015579\_contig\_number\_15087SRS016018\_contig\_number\_10573SRS016095\_contig\_number\_11388SRS016132\_contig\_number\_1243SRS016203\_contig\_number\_2898SRS016495\_contig\_number\_contig-100\_522.113732SRS017191\_contig\_number\_15963SRS017701\_contig\_number\_12282SRS017859\_contig\_number\_contig-100\_388.36426SRS018427\_contig\_number\_12943SRS018427\_contig\_number\_contig-100\_614.156173SRS018656\_contig\_number\_12006SRS019068\_contig\_number\_35875SRS019161\_contig\_number\_6197SRS019267\_contig\_number\_1455SRS019601\_contig\_number\_11300SRS019685\_contig\_number\_35676SRS019787\_contig\_number\_675SRS024075\_contig\_number\_11665SRS043411\_contig\_number\_1267SRS043701\_contig\_number\_1355SRS045713\_contig\_number\_13586SRS047014\_contig\_number\_32985SRS048060\_contig\_number\_2071SRS048870\_contig\_number\_12206SRS049446\_contig\_number\_3463SRS049446\_contig\_number\_11266SRS050299\_contig\_number\_4295SRS052697\_contig\_number\_18652SRS053214\_contig\_number\_21143SRS053398\_contig\_number\_16892SRS054590\_contig\_number\_469SRS054905\_contig\_number\_16620SRS058070\_contig\_number\_20831SRS058770\_contig\_number\_24138SRS062701\_contig\_number\_5689SRS063040\_contig\_number\_39281SRS065504\_contig\_number\_38449SRS075341\_contig\_number\_3542SRS075878\_contig\_number\_18104SRS077086\_contig\_number\_243SRS077335\_contig\_number\_1302SRS077730\_contig\_number\_78SRS077730\_contig\_number\_17532SRS097889\_contig\_number\_15116SRS097920\_contig\_number\_23143SRS098073\_contig\_number\_2750SRS1041031\_contig\_number\_6842SRS1041038\_contig\_number\_14664SRS1041092\_contig\_number\_8512SRS1041118\_contig\_number\_7659SRS1041132\_contig\_number\_4073SRS1041133\_contig\_number\_21296SRS1041136\_contig\_number\_12060SRS1041141\_contig\_number\_11024SRS1041142\_contig\_number\_1150SRS104400\_contig\_number\_25291SRS104975\_contig\_number\_3027SRS104975\_contig\_number\_18770SRS1055038\_contig\_number\_999SRS140513\_contig\_number\_9712SRS140513\_contig\_number\_15812SRS142503\_contig\_number\_19127SRS142712\_contig\_number\_4868SRS142923\_contig\_number\_13583SRS143342\_contig\_number\_17991SRS143780\_contig\_number\_4394SRS144183\_contig\_number\_2406SRS148424\_contig\_number\_18637SRS148511\_contig\_number\_20274SRS148784\_contig\_number\_14203SRS148817\_contig\_number\_10668SRS149325\_contig\_number\_11513SRS893172\_contig\_number\_2025SRS893187\_contig\_number\_1290SRS893187\_contig\_number\_12004SRS893230\_contig\_number\_7865SRS893358\_contig\_number\_12550SRS893373\_contig\_number\_21382SRS971275\_contig\_number\_14140SRS971275\_contig\_number\_43420


SRS145497\_contig\_number\_2865SRS893369\_contig\_number\_8269
2
species
28116

1

SRS1055043\_contig\_number\_20737
1917883
species

species
626929

SRS016267\_contig\_number\_7281SRS075878\_contig\_number\_920SRS077335\_contig\_number\_1428
3

162156
species

SRS018984\_contig\_number\_26699SRS019445\_contig\_number\_26670SRS024492\_contig\_number\_16283SRS053649\_contig\_number\_19842SRS077127\_contig\_number\_2038SRS147346\_contig\_number\_34146
6

1

SRS024075\_contig\_number\_8842
species
93975

1

SRS144506\_contig\_number\_43360
1929999
species


SRS050925\_contig\_number\_23012
1
species
290053


SRS1055099\_contig\_number\_4379SRS893342\_contig\_number\_2925SRS971275\_contig\_number\_23169
3
817
species

19

SRS012273\_contig\_number\_15512SRS013940\_contig\_number\_8776SRS013940\_contig\_number\_20992SRS014948\_contig\_number\_3316SRS015960\_contig\_number\_11979SRS016629\_contig\_number\_2910SRS018623\_contig\_number\_15623SRS018836\_contig\_number\_1772SRS045645\_contig\_number\_3056SRS049995\_contig\_number\_15378SRS056259\_contig\_number\_22226SRS075078\_contig\_number\_23246SRS076756\_contig\_number\_13308SRS076976\_contig\_number\_20121SRS101376\_contig\_number\_20496SRS104636\_contig\_number\_30628SRS142599\_contig\_number\_15471SRS144362\_contig\_number\_12733SRS144537\_contig\_number\_46158
329854
species


SRS019787\_contig\_number\_392
1
1263039
species


SRS063489\_contig\_number\_7054
1
species
821


SRS011084\_contig\_number\_21915SRS011302\_contig\_number\_22190SRS012273\_contig\_number\_25550SRS013158\_contig\_number\_634SRS013382\_contig\_number\_192SRS013521\_contig\_number\_4290SRS013521\_contig\_number\_contig-100\_1973.81601SRS013638\_contig\_number\_16112SRS013687\_contig\_number\_22561SRS014412\_contig\_number\_18831SRS014683\_contig\_number\_5870SRS014855\_contig\_number\_11316SRS014923\_contig\_number\_20172SRS015431\_contig\_number\_21911SRS015579\_contig\_number\_13460SRS015663\_contig\_number\_21347SRS015694\_contig\_number\_4549SRS015782\_contig\_number\_14054SRS015854\_contig\_number\_4763SRS016056\_contig\_number\_2157SRS016335\_contig\_number\_26511SRS016629\_contig\_number\_3871SRS017247\_contig\_number\_3917SRS017521\_contig\_number\_19683SRS017701\_contig\_number\_19825SRS017745\_contig\_number\_626SRS017745\_contig\_number\_1117SRS017859\_contig\_number\_1404SRS018133\_contig\_number\_4168SRS018817\_contig\_number\_17639SRS018836\_contig\_number\_19988SRS018888\_contig\_number\_7132SRS018936\_contig\_number\_594SRS019030\_contig\_number\_11288SRS019068\_contig\_number\_9449SRS019178\_contig\_number\_1582SRS019381\_contig\_number\_4229SRS019397\_contig\_number\_2057SRS019685\_contig\_number\_24891SRS020271\_contig\_number\_contig-100\_1231.24995SRS020394\_contig\_number\_11086SRS020869\_contig\_number\_13141SRS022071\_contig\_number\_13729SRS022137\_contig\_number\_3316SRS022713\_contig\_number\_146SRS023583\_contig\_number\_4842SRS023715\_contig\_number\_24855SRS023971\_contig\_number\_4256SRS024075\_contig\_number\_46SRS024388\_contig\_number\_9347SRS042966\_contig\_number\_1781SRS043001\_contig\_number\_1840SRS043768\_contig\_number\_2394SRS045645\_contig\_number\_3216SRS045739\_contig\_number\_6528SRS045826\_contig\_number\_11921SRS046369\_contig\_number\_9560SRS046717\_contig\_number\_646SRS047014\_contig\_number\_13437SRS047433\_contig\_number\_13574SRS048164\_contig\_number\_12924SRS049402\_contig\_number\_4499SRS049712\_contig\_number\_4333SRS049773\_contig\_number\_12625SRS049896\_contig\_number\_9818SRS050026\_contig\_number\_1101SRS050520\_contig\_number\_324SRS050752\_contig\_number\_6110SRS050925\_contig\_number\_4587SRS050941\_contig\_number\_11046SRS051031\_contig\_number\_3971SRS052697\_contig\_number\_1611SRS055982\_contig\_number\_5137SRS057478\_contig\_number\_66SRS057480\_contig\_number\_10346SRS058770\_contig\_number\_3458SRS062654\_contig\_number\_6187SRS064645\_contig\_number\_1353SRS064645\_contig\_number\_1424SRS064757\_contig\_number\_16102SRS065176\_contig\_number\_1465SRS065397\_contig\_number\_2667SRS065397\_contig\_number\_9418SRS074670\_contig\_number\_2813SRS075773\_contig\_number\_20017SRS075821\_contig\_number\_4966SRS075984\_contig\_number\_8927SRS076756\_contig\_number\_1098SRS076804\_contig\_number\_125SRS076876\_contig\_number\_446SRS076976\_contig\_number\_12056SRS077454\_contig\_number\_12223SRS077552\_contig\_number\_2475SRS078176\_contig\_number\_9263SRS078665\_contig\_number\_6882SRS097958\_contig\_number\_22481SRS098061\_contig\_number\_1906SRS098881\_contig\_number\_334SRS100021\_contig\_number\_1443SRS104036\_contig\_number\_contig-100\_1365.41150SRS1041036\_contig\_number\_1400SRS1041037\_contig\_number\_962SRS1041039\_contig\_number\_19413SRS1041095\_contig\_number\_1277SRS1041130\_contig\_number\_884SRS1041132\_contig\_number\_2221SRS1041137\_contig\_number\_13531SRS1041139\_contig\_number\_534SRS1041143\_contig\_number\_6985SRS1041144\_contig\_number\_6377SRS1041147\_contig\_number\_299SRS1041157\_contig\_number\_4051SRS104311\_contig\_number\_24338SRS104327\_contig\_number\_341SRS104400\_contig\_number\_14406SRS104912\_contig\_number\_13324SRS105082\_contig\_number\_10778SRS105153\_contig\_number\_1338SRS1055069\_contig\_number\_9436SRS1055076\_contig\_number\_405SRS140492\_contig\_number\_28711SRS142542\_contig\_number\_1893SRS143070\_contig\_number\_4043SRS143148\_contig\_number\_13825SRS143372\_contig\_number\_4580SRS143417\_contig\_number\_2302SRS143523\_contig\_number\_1388SRS143598\_contig\_number\_1344SRS143722\_contig\_number\_3978SRS143876\_contig\_number\_22966SRS143991\_contig\_number\_2620SRS144135\_contig\_number\_36132SRS144297\_contig\_number\_9938SRS144506\_contig\_number\_47213SRS144714\_contig\_number\_25439SRS146764\_contig\_number\_10115SRS146813\_contig\_number\_4442SRS147039\_contig\_number\_214SRS147271\_contig\_number\_22671SRS147425\_contig\_number\_9036SRS147614\_contig\_number\_1759SRS148196\_contig\_number\_27836SRS148253\_contig\_number\_225SRS148511\_contig\_number\_8975SRS148721\_contig\_number\_17250SRS148784\_contig\_number\_22809SRS149075\_contig\_number\_15286SRS149181\_contig\_number\_20996SRS893172\_contig\_number\_8151SRS893187\_contig\_number\_11771SRS893259\_contig\_number\_3459SRS893279\_contig\_number\_10900SRS893288\_contig\_number\_1313SRS893327\_contig\_number\_8958SRS893341\_contig\_number\_2249SRS893358\_contig\_number\_9178SRS893378\_contig\_number\_14865SRS893380\_contig\_number\_1102SRS893382\_contig\_number\_1300
159
46506
species


SRS016989\_contig\_number\_10511SRS024492\_contig\_number\_18154
2
820
species
